# Comparison of the Fluid Resuscitation Rate with and without External Pressure Using Two Intraosseous Infusion Systems for Adult Emergencies, the CITRIN (Comparison of InTRaosseous infusion systems in emergency medicINe)-Study

**DOI:** 10.1371/journal.pone.0143726

**Published:** 2015-12-02

**Authors:** Niels Hammer, Robert Möbius, André Gries, Björn Hossfeld, Ingo Bechmann, Michael Bernhard

**Affiliations:** 1 Department of Anatomy, University of Otago, Dunedin, New Zealand; 2 Institute of Anatomy, University of Leipzig, Faculty of Medicine, Leipzig, Germany; 3 Emergency Department, University Hospital of Leipzig, Leipzig, Germany; 4 Department of Anaesthesiology and Intensive Care Medicine, Section Emergency Medicine, Federal Armed Forces Medical Hospital, Ulm, Germany; Georgia Regents University, UNITED STATES

## Abstract

**Introduction:**

Intraosseous infusion is recommended if peripheral venous access fails for cardiopulmonary resuscitation or other medical emergencies. The aim of this study, using body donors, was to compare a semi-automatic (EZ-IO^®^) device at two insertion sites and a sternal intraosseous infusion device (FASTR^™^).

**Methods:**

Twenty-seven medical students being inexperienced first-time users were randomized into three groups using EZ-IO and FASTR. The following data were evaluated: attempts required for successful placement, insertion time and flow rates with and without external pressure to the infusion.

**Results:**

The first-pass insertion success of the EZ-IO tibia, EZ-IO humerus and FASTR was 91%, 77%, and 95%, respectively. Insertion times (MW±SD) did not show significant differences with 17±7 (EZ-IO tibia) vs. 29±42 (EZ-IO humerus) vs. 33±21 (FASTR), respectively. One-minute flow rates using external pressures between 0 mmHg and 300 mmHg ranged between 27±5 to 69±54 ml/min (EZ-IO tibia), 16±3 to 60±44 ml/min (EZ-IO humerus) and 53±2 to 112±47 ml/min (FASTR), respectively. Concerning pressure-related increases in flow rates, negligible correlations were found for the EZ-IO tibia in all time frames (c = 0.107–0.366; p≤0.013), moderate positive correlations were found for the EZ-IO humerus after 5 minutes (c = 0.489; p = 0.021) and strong positive correlations were found for the FASTR in all time frames (c = 0.63–0.80; p≤0.007). Post-hoc statistical power was 0.62 with the given sample size.

**Conclusions:**

The experiments with first-time users applying EZ-IO and FASTR in body donors indicate that both devices may be effective intraosseous infusion devices, likely suitable for fluid resuscitation using a pressure bag. Variations in flow rate may limit their reliability. Larger sample sizes will prospectively be required to substantiate our findings.

## Introduction

Intraosseous access is recommended by the current guidelines of the European Resuscitation Council (ERC) and other organizations for pediatric and adult patients, if establishing peripheral venous access is time consuming or impossible for cardiopulmonary resuscitation (CPR) or other emergencies [1–4]. Current studies have shown that an intraosseous access is safe, simple, effective and associated with a low complication rate [5].

The development of new devices increased the available options for vascular access through the intraosseous route, particularly with the tibia or humerus as puncture site in both pediatric and adult patients (e.g. EZ-IO^®^, Teleflex Medical, United States) or the sternal approach in patients older than 12 years (e.g. FASTR; FASTResponder^™^ Intraosseous Device, PYNG Medical, Canada). Nevertheless, little evidence exists on the fluid resuscitation rate of different intraosseous infusion systems in adult emergency patients: The low fluid rate through these devices remains an area of debate and infusion rates between 200 and 9900 ml/h were reported according to external use of pressure bags [6–12]. The information provided by manufacturers' instruction manuals ranges from 30 ml/h to 120 ml/min (without pressure and with pressure) using the FASTR. Lower infusion rates ranging between 16 vs. 83 ml/min (without pressure vs. with pressure) have been described for the semi-automatic EZ-IO tibia and humerus infusion systems [7]. However, most of the existing studies are based on animal models. Furthermore, studies on the variations in the given flow rates are missing. Such data could help estimate the reliability of the different devices for fluid resuscitation therapy.

This experimental study aimed to investigate the time- and pressure-dependent flow rates following intraosseous puncture of human body donors at three different anatomic sites by inexperienced first-time users.

## Methods

The ethics committee of the Medical Faculty of the University of Leipzig, Germany approved the CITRIN study protocol (no. 141-15-20042-15).

### Study design, data collection and analysis

Two different devices were applied at three different anatomical sites: The semi-automatic EZ-IO (EZ-IO^®^, Teleflex Medical, USA) consists of a multi-use, non-rechargeable battery-powered driver with integrated beveled, hollow drill-tipped needles. The depth of the needle is not generally determined by means of “loss-of-resistance” in this technique but rather the needle length specified the individual puncture depth [13]. To establish the intraosseous access in an adult (>39 kg body weight), the recommended needle length is 25 mm in total for the proximal tibia. For the proximal humerus, the recommended needle length is 45 mm. The outside diameter of the needles is 15 G (1.8 mm). The manufacturer’s instruction manual reports infusion rates of 16 ml/min without pressure and 165 ml/min with pressure at both puncture sites.

The FASTR (FASTResponder^™^ Intraosseous Device, PYNG Medical, Canada) consists of a single-use, non-electric and manual device with an integrated intraosseous needle. The position of the needle is determined at 15 mm below the supra-sternal notch and penetrates 6 mm into the manubrium [14]. The manufacturer’s instruction manual reports infusion rates of 38 ml/min without additional pressure and 94 ml/min with a pressure of 300 mmHg.

Volumes were derived from the mass changes of the infusion bags filled with normal saline in the respective time frames, recorded with a precision scale (Kern PEJ; Kern & Sohn GmbH, Balingen, Germany). The vertical distance between the infusion bag and the heart level of the donors was standardized to 800 mm. The resulting fluid column produced a hydrostatic pressure of 59 mmHg.

### Body donors and study participants

The experiments were performed with 27 body donors (S1 Table) as a model to simulate intraosseous infusion attempts in a next to real circumstances like an emergency setting. Before they passed away, all body donors gave their informed and written consent to the donation of their bodies for teaching and research purposes. Being part of the body donor program, regulated by the Saxonian Death and Funeral Act of 1994 (third section, paragraph 18 item 8), institutional approval for the use of the post-mortem tissues was obtained from the Institute of Anatomy, University of Leipzig. Three donors were used in an anatomically unfixed condition, whereas the remaining 24 donors were fixed with ethanol-glycerin [15,16]. All donors underwent X-ray imaging of the thorax, both humeri and tibiae. Anatomical sites with implants or fractures were excluded as puncture sites.

Twenty-seven second and forth year medical students (mean age 22.7 ± 4.1 years, 16 ♂, 11 ♀ S2 Table) of the University of Leipzig were enrolled in this study on a voluntarily basis. Only first-time users without prior experience in the use of any of both intraosseous devices were admitted. All participants gave their written consent to participate prior to the study. The intraosseous devices were described theoretically and practically in models in a standardized 15-minute lecture held individually to each student. The theory included the respective indications, complications and the performance of each system. Thereafter, the students performed in random order each of the intraosseous punctures in the ethanol-fixed donors under supervision of one of the principal investigators.

The number of body donors and participants was limited to 27 each due to financial constraints concerning the devices and body donation-related costs and to body donor availability. Presumably the given sample size influenced the statistical power of the findings.

### Flow rates in fresh body donors under different pressure levels

Three anatomically unfixed body donors were used for determining the flow rates of the EZ-IO humerus, tibia and FASTR at different pressure levels. The intraosseous systems were inserted into the humerus, tibia and sternum according to the manufacturers’ recommendations. Following a flush of 10 ml normal saline, flow rates were recorded in time frames of 1, 3 and 5 minutes without applying additional pressure to the infusion device and with an (additional) external pressure of 50 to 300 mmHg increased at 50-mmHg increments.

### Flow rates and evaluation of the intraosseous systems by technically inexperienced participants

Given the limited number of devices and body donors, some of the students were restricted to applying either the EZ-IO or the FASTR devices (S3 Table **)**. The sequence at which the punctures were carried out by the participants, and the body donors on which the punctures were performed, were randomized for each participant to minimize any bias related to learning effects. Consequently, each of the devices was used as the first one in 1/3 of all cases and as the last one in 1/3 of all cases. There were three different participant groups with the following sequences: group 1: FASTR, (EZ-IO humerus, EZ-IO tibia or EZ-IO tibia, EZ-IO humerus), n = 7; group 2: EZ-IO tibia, EZ-IO humerus, (FASTR), n = 10; group 3: EZ-IO humerus, (FASTR), EZ-IO tibia, n = 10. A total of 22 EZ-IO tibia, 22 EZ-IO humerus and 19 FASTR devices were utilized. The data were collected using evaluation forms, including donors’ age, gender, body weight, length and body-mass index (BMI) and participants’ age, gender, semester. The insertion times for each device and puncture site were recorded. Insertion time was defined as the time needed to identify the anatomical site, pick up and apply the intraosseous infusion system and to insert the needle into the bone. Furthermore, the number of attempts required to place the needle successfully was recorded. The success of the puncture was checked by means of a 10-ml flush of normal saline into the bone cavity under permanent visual and haptic control. Visual deformation of the covering skin or leakage from the puncture site was considered as an unsuccessful attempt. Each participant was allowed a maximum of two attempts to establish the access in case of the EZ-IO and one attempt in case of the FASTR. Immediately following the flush, the infusion rates were measured in 1-, 3- and 5-minute time frames without additional pressure and with a pressure of 150 mmHg. The participants graded the devices and the principal investigators graded the participants using a six-point Likert scale (1 = excellent / grade A+ to 6 = non-satisfactory / grade F). Also, a relative ranking of the devices and technical complications were documented (e.g. needle breakage, bent needle, defective batteries, bone fracturing, paravasation)[13].

### Statistical analyses

All data were digitalized and evaluated statistically, using Microsoft Excel version 2013 (Microsoft Corporation, Redmond, WA, USA) and SPSS version 22.0 (Chicago, IL, USA). The results are presented as (absolute) mean values±standard deviations or as percentages. The Kolmogorov-Smirnov test was applied to check normal distribution of the data. Consequently the Student's t-test, the Mann-Whitney U-test or the one-way ANOVA test with post-hoc analysis was applied. Correlations between the flow rates and infusion time or BMI were determined using the Pearson test. G*Power version 3.1.9.2 was used for the power analyses. *P* values of 0.05 or less were considered to be statistically significant.

## Results

### Participant and body donor characteristics

Twenty-seven participants applied the EZ-IO devices to the tibiae and humeri and FASTR devices to sternums of 27 body donors. Post-hoc analyses revealed a power of 0.62 on basis of the given sample size. The participant and body donor characteristics were similar and non-significantly different for the three groups (S1 and S2 Tables). Fixed and unfixed donors were similar and non-significantly different in age, gender, body weight and BMI with the only exception of body length.

### User friendliness

**Fig 1:**
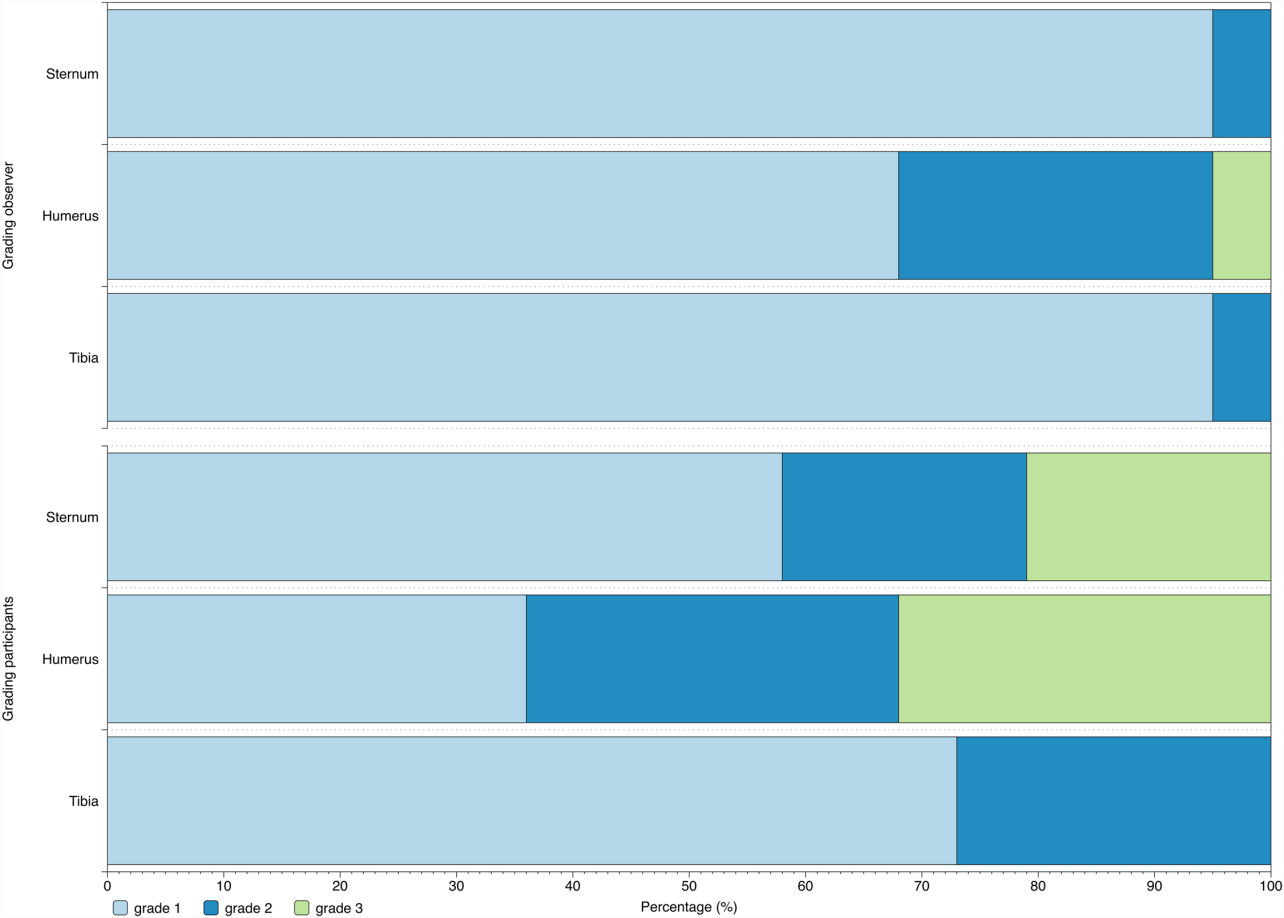
Participant and observer grading on the EZ-IO tibia, EZ-IO humerus and FASTR device.

Concerning the consecutive order of device popularity, the EZ-IO tibia was rated by the participants to be the most popular of the three devices among the majority of applicants (62%) followed by the FASTR (38%). The least popular device was the EZ-IO humerus. Participant ratings were significantly better for the EZ-IO tibia (1.3±0.5) than for the EZ-IO humerus (2.0±0.8; p = 0.006; Fig 1;Table 1). The FASTR (1.6±0.8) was rated non-significantly lower than the EZ-IO tibia (p = 0.349) and non-significantly better than the EZ-IO humerus (p = 0.390). Observer ratings of the participants were significantly lower for the use of the EZ-IO humerus (1.1±0.2) than for the use of the EZ-IO tibia (1.0±0.2; p = 0.025) and the use of the FASTR (1.4±0.6; p = 0.039; Fig 1; Table 1).

**Table 1:** Participant grading, insertion time and number of attempts using the EZ-IO tibia, EZ-IO humerus and FASTR devices.

Participants	Grading							Insertion time			Attempt	
	[1 = excellent to 6 = failure]							[sec]			[number]	
	Participant			Observer								
	EZ-IO tibia	EZ-IO humerus	FASTR	EZ-IO Tibia	EZ-IO humerus	FASTR	EZ-IO tibia	EZ-IO humerus	FASTR	EZ-IO tibia	EZ-IO humerus	FASTR
**Mean value**	1.3	2.0	1.6	1.0	1.4	1.1	17.0	29.1	32.6	1.1	1.2	1.1
**Standard deviation**	0.5	0.8	0.8	0.2	0.6	0.2	7.2	42.3	20.6	0.3	0.4	0.2
**Median**	1	2	1	1	1	1	15	19	30	1	1	1
**Minimum**	1	1	1	1	1	1	8	5	14	1	1	1
**Maximum**	2	3	3	2	3	2	33	210	110	2	2	2
***p value (ANOVA)***		*0*.*008*			*0*.*012*			*0*.*165*			*0*.*209*	
***post hoc analysis (if applicable)***												
*EZ-IO tibia vs*. *EZ-IO humerus*		*0*.*006*			*0*.*025*							
*EZ-IO humerus vs*. *FASTR*		*0*.*390*			*0*.*039*							
*EZ-IO tibia vs*. *EZ-IO humerus*		*0*.*349*			*1*.*0*							

### Insertion time

The least insertion times and the least variations in insertion time were observed for the EZ-IO tibia (17±7 sec), followed by the EZ-IO humerus (29±42 sec) and the FASTR (33±21 sec) without any significant differences (Table 1). The first-pass insertion success rates of the EZ-IO tibia, humerus and the FASTR were 91% (20/22), 77% (17/22), and 95% (18/19), respectively. A second insertion attempt for the successful placement of the EZ-IO tibia and humerus were needed in two (9%) and five cases (23%), respectively.

### Complication rate

In the EZ-IO tibia, complications were observed in 2/22 cases (lacking flow in one case caused by bent needle; S3 Table), in 5/22 cases in the EZ-IO humerus (lacking flow) and in 5/19 cases with the FASTR (lacking flow in two cases caused by failure of the needle-securing mechanism in three cases).

### Flow rates in unfixed body donors

**Fig 2:**
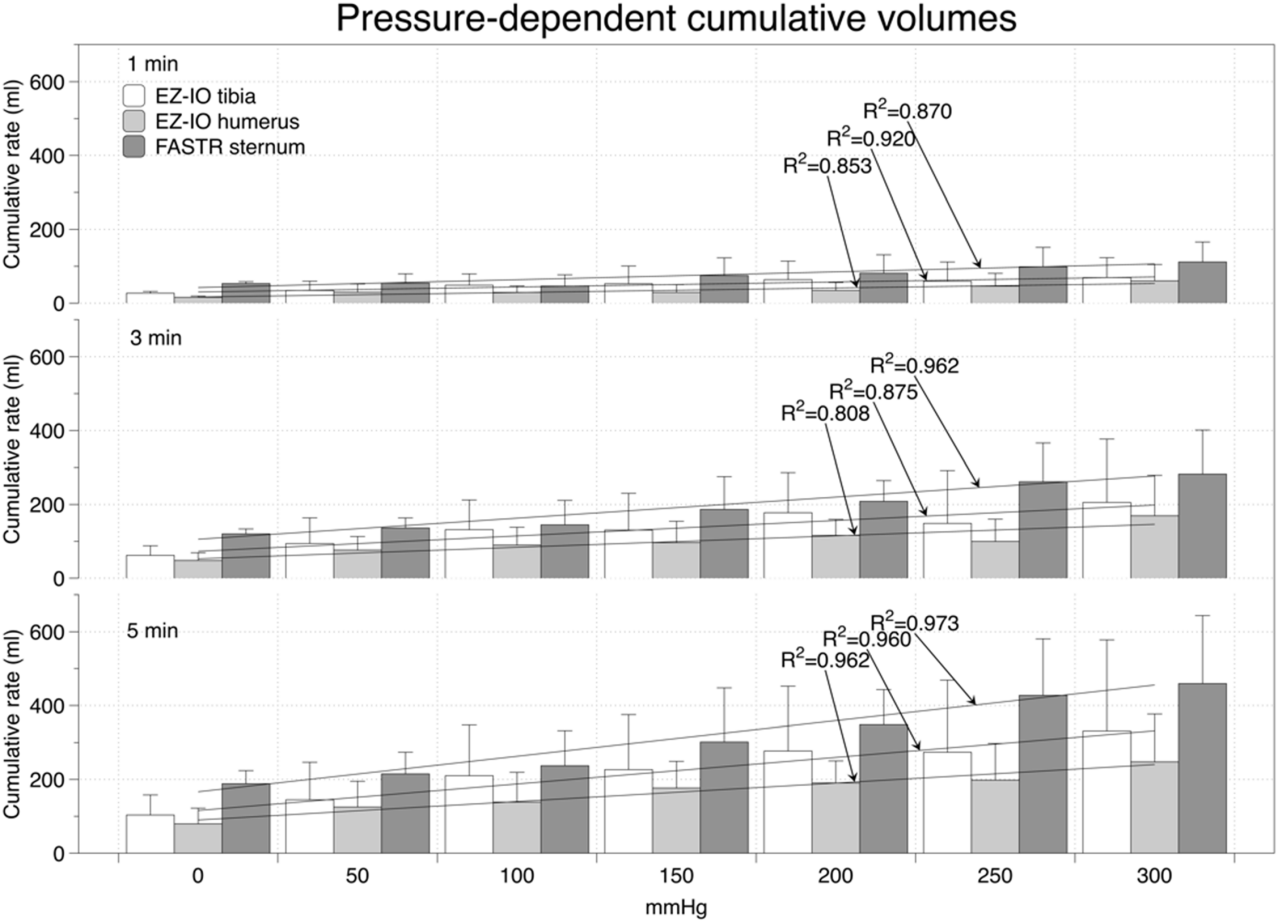
Pressure-dependent cumulative volumes in unfixed donors using the EZ-IO tibia, EZ-IO humerus and FASTR device.

**Fig 3:**
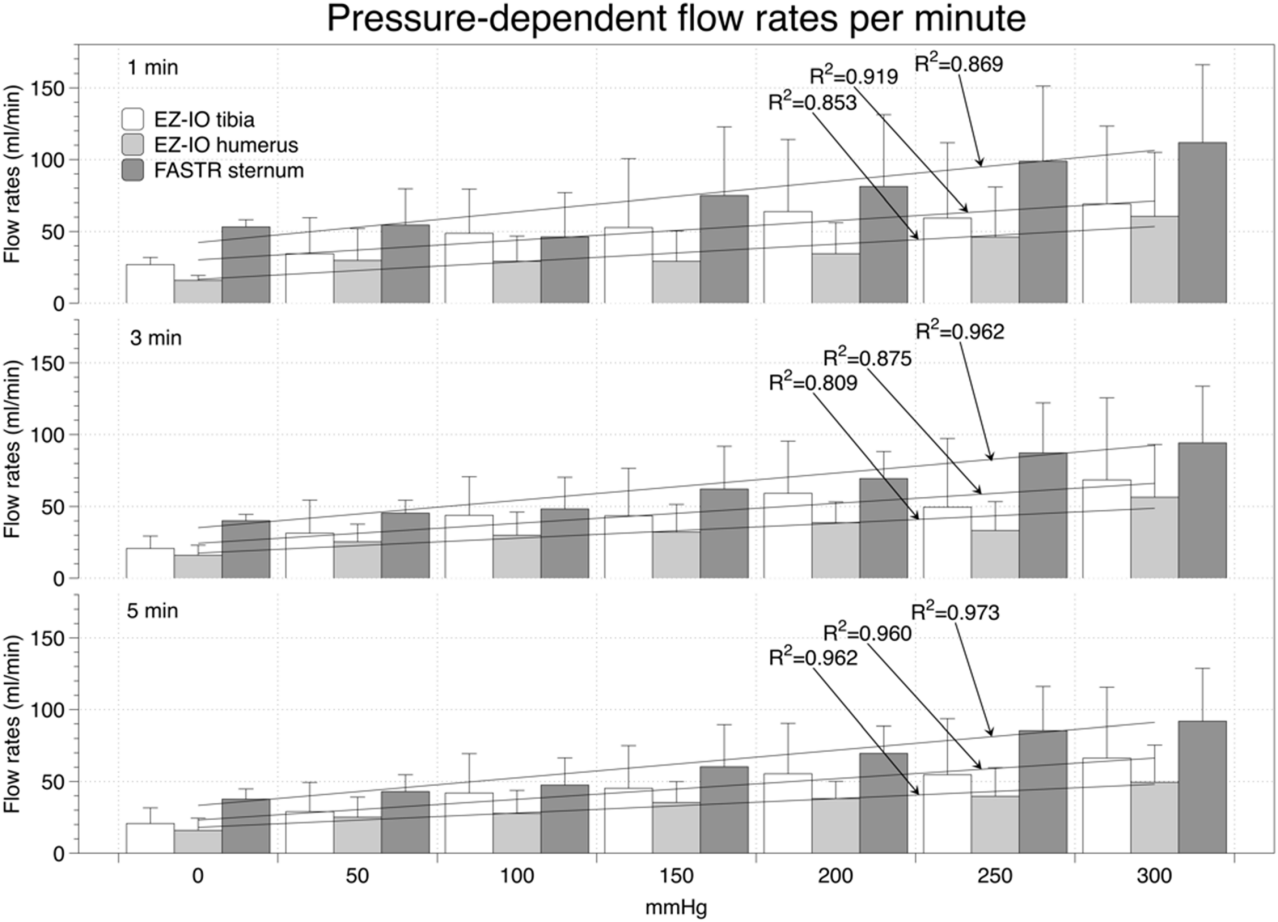
Pressure-dependent flow rates per minute in unfixed donors using the EZ-IO tibia, EZ-IO humerus and FASTR device.

In the unfixed body donors, the cumulative volumes and flow rates were pressure-dependent and increased in the three devices in 50-mmHg increments (Figs 2 and 3; S4 and S5 Tables). At each of the given pressure levels and time frames, cumulative flow rates at the sternum were highest (Fig 2). Flow rates in the humerus were lower than in the tibia, but without reaching a statistical significance. One-minute flow rates ranged between 27±5 and 69±54 ml/min for the EZ-IO tibia, 16±3 and 60±44 ml/min for the EZ-IO humerus and between 53±2 and 112±47 ml/min for the FASTR between 0 mmHg and 300 mmHg, respectively (Fig 3). The mean flow rates per three-minute interval between 0 and 300 mmHg ranged from 21±9 to 69±57 ml/min for the EZ-IO tibia, from 16±7 to 56±37 ml/min for the EZ-IO humerus and from 40±4 to 94±40 ml/min for the FASTR, respectively. After 5 minutes, mean flow rates decreased for the devices: EZ-IO tibia with 21±11 to 66±49 ml/min and for the FASTR with 38±7 to 92±37 ml/min between 0 and 300 mmHg, respectively. Five-minute flow rates in the EZ-IO humerus increased slightly with 16±9 to 50±26 ml/min between 0 and 300 mmHg, respectively. Strong positive correlations were found for the pressure-dependent flow rates in all devices (c≥0.932; p≤0.001). No correlations were found for the flow rates and age of body donors, body length and weight or BMI in the fresh donors.

### Flow rates in fixed body donors

**Fig 4:**
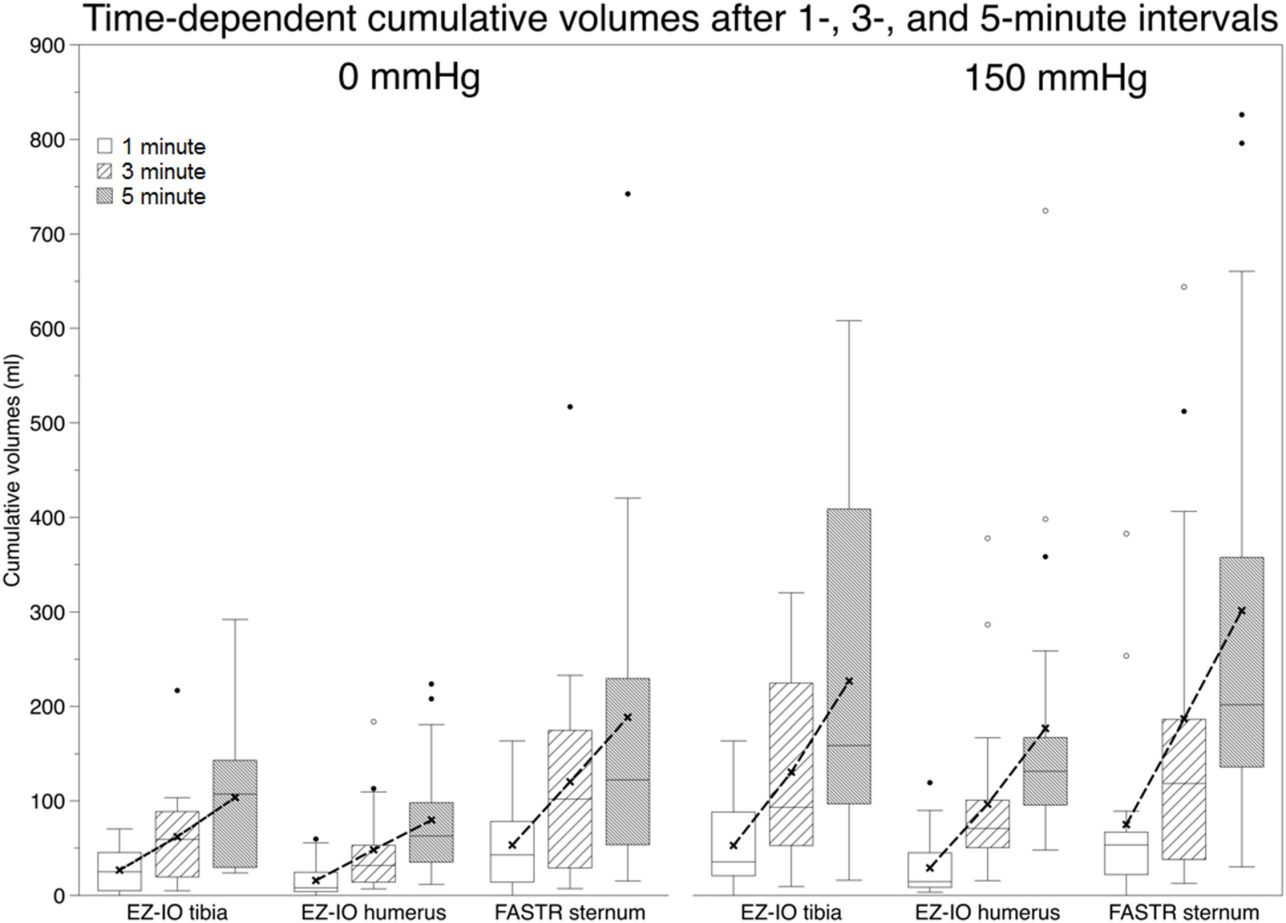
Time-dependent cumulative volumes after 1-, 3- and 5-minute intervals using the EZ-IO tibia, EZ-IO humerus and FASTR device. Box plot = 25–75% percentile, whiskers = 1,5 x standard deviation, points = outliers. white box = 1 minute, pattern box = 3 minutes, grey box = 5 minutes.

**Fig 5:**
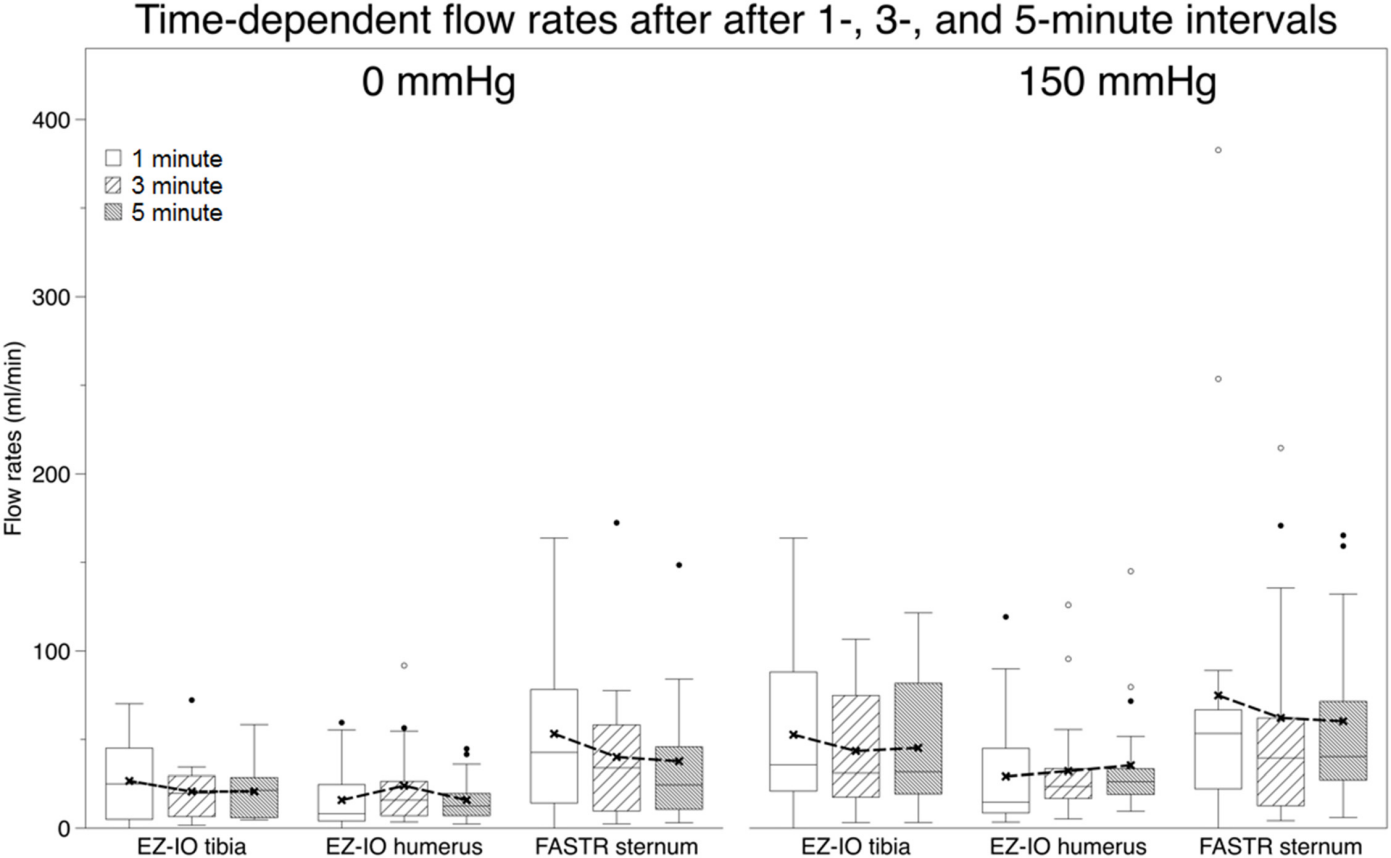
Time-dependent flow rates after 1-, 3- and 5-minute intervals using the EZ-IO tibia, EZ-IO humerus and FASTR device. Box plot = 25–75% percentile, whiskers = 1,5 x standard deviation, points = outliers. white box = 1 minute, pattern box = 3 minutes, grey box = 5 minutes.

In the experiments performed with the ethanol-fixed body donors, cumulative volumes and flow rates were obtained as given in S6 and S7 Tables. There was a marked difference between the infusion with 0 mmHg and with 150 mmHg. Flow rates and cumulative volumes were larger using the FASTR, compared to the EZ-IO humerus and the EZ-IO tibia, but not differing significantly at the respective time frames and pressures. Inner comparison showed that in each intraosseous device the time-dependent cumulative volumes increased significantly (p≤0.001). However, in line with the flow rates in the unfixed donors, flow rates decreased time-dependently for the EZ-IO tibia and FASTR. Using the EZ-IO humerus, time-dependent flow rates tended to slightly increase. For the pressure-related increase in flow rates, negligible correlations were found for the EZ-IO tibia in all time frames (correlation coefficient = c = -0.107 to 0.366; p≤0.013), moderate positive correlations were found for the EZ-IO humerus after 5 minutes (c = 0.489; p = 0.021) and strong positive correlations were found for the FASTR in all time frames (c = 0.63 to 0.80; p≤0.007). No correlations were found for the flow rates and donors’ age, body length and weight or BMI in the ethanol-fixed donors in line with the unfixed donors. The cumulative volumes and flow rates of the fixed donors were adjusted to the data of the unfixed donors. For this purpose, a normalizing ratio was computed for each device and time frame in the 0- and 150-mmHg pressure level. The resulting flow rates and variations are given in Tables 2 and 3 and Figs 4 and 5. Cumulative flow rates and flow rates per minute varied the most in the FASTR at 150 mmHg (variation coefficient 0.85 to 1.31), followed by the EZ-IO humerus at 0 mmHg (variation coefficient 0.80–1.09), respectively. The smallest variation in flow rate was observed in the EZ-IO tibia.

**Table 2:** Normalized cumulative volumes in ethanol-fixed body donors.

Pressure		EZ-IO tibia [ml] at min			EZ-IO humerus [ml] at min			FASTR [ml] at min		
[mmHg]		1	3	5	1	3	5	1	3	5
**0**	**Mean value**	26.9	62.0	103.5	15.9	48.1	79.6	53.2	120.3	188.5
	**Standard deviation**	22.4	49.6	75.9	17.4	45.2	63.6	49.2	125.2	188.0
	***variation coefficient***	0.83	0.80	0.73	1.09	0.94	0.80	0.93	1.04	1.00
	**Minimum**	0.0	4.9	23.8	0.0	7.1	11.8	0.0	7.3	15.3
	**Maximum**	70.2	216.8	292.0	59.5	183.7	223.6	163.7	517.0	742.2
	***p value (inter group)***	*0*.*447 – 0*.*616*								
**150**	**Mean value**	52.7	130.5	226.7	29.2	96.5	176.8	74.9	186.7	301.6
	**Standard deviation**	46.6	99.3	173.3	30.7	87.5	151.5	98.4	182.0	255.1
	***variation coefficient***	*0*.*89*	*0*.*76*	*0*.*76*	*1*.*05*	*0*.*91*	*0*.*86*	*1*.*31*	*0*.*97*	*0*.*85*
	**Minimum**	0.0	9.6	16.1	3.5	15.7	47.8	0.0	12.7	30.2
	**Maximum**	163.7	320.2	608.0	119.3	377.9	724.7	382.7	643.9	826.2
	***p value (inter group)***	*0*.*222 – 0*.*643*								

**Table 3:** Normalized flow rates in ethanol-fixed body donors.

Pressure		EZ-IO tibia [ml/min] at min			EZ-IO humerus [ml/min] at min			FASTR [ml/min] at min		
**[mmHg]**		**1**	**3**	**5**	**1**	**3**	**5**	**1**	**3**	**5**
**0**	**Mean value**	26.9	20.7	20.7	15.9	24.0	15.9	53.2	40.1	37.7
	**Standard deviation**	22.4	16.5	15.2	17.4	22.6	12.7	49.2	41.7	37.6
	***variation coefficient***	*0*.*83*	*0*.*80*	*0*.*73*	*1*.*09*	*0*.*94*	*0*.*80*	*0*.*93*	*1*.*04*	*1*.*00*
	**Minimum**	0.0	1.6	4.8	0.0	3.5	2.4	0.0	2.4	3.1
	**Maximum**	70.2	72.3	58.4	59.5	91.8	44.7	163.7	172.3	148.4
	***p value (inter group)***	*0*.*446 – 0*.*615*								
**150**	**Mean value**	52.7	43.5	45.3	29.2	32.2	35.4	74.9	62.2	60.3
	**Standard deviation**	46.6	33.1	34.7	30.7	29.2	30.3	98.4	60.7	51.0
	***variation coefficient***	*0*.*89*	*0*.*76*	*0*.*76*	*1*.*05*	*0*.*91*	*0*.*86*	*1*.*31*	*0*.*97*	*0*.*85*
	**Minimum**	0.0	3.2	3.2	3.5	5.2	9.6	0.0	4.2	6.0
	**Maximum**	163.7	106.7	121.6	119.3	126.0	144.9	382.7	214.6	165.2
	***p value (inter group)***	*0*.*222 – 0*.*643*								

## Discussion

Intravenous vascular access is often difficult to achieve in critically ill patients. Moreover, central line placement can be time consuming [17]. The given three intraosseous devices may be regarded as commonly used in pre-hospital and in-hospital emergency settings to infuse drugs, fluids and blood products [18,19].

In our experiments with unfixed body donors, similar flow rates were observed as recently found in clinical [8] and experimental studies [20]. Comparison of the flow rates and cumulative volumes of the unfixed to ethanol-fixed donors revealed different flow rates but similar relative standard deviations, indicating that the ethanol-fixed donors are a valid model to investigate intraosseous devices in a safe environment for inexperienced performers. Using the respective devices with a pressure bag applying 300 mmHg, initial flow rates of >100 ml/min were achieved in the first minute using the FASTR, and ≥60 ml/min using the EZ-IO tibia and humerus (Fig 3), each after a initial application of 10-ml bolus of normal saline. The values at 0 mmHg were less than 50% of the rates at 300 mmHg on the pressure bag. The given flow rates and cumulative volumes were similar to the data of Pasley et al. [20], who applied the FAST1 system (PYNG Medical, Canada), the EZ-IO humerus and EZ-IO tibia to soft-embalmed donors. Significant differences between the EZ-IO humerus and tibia were previously found clinically [21] and experimentally in human donors [20] and porcine models [11]. These differences could not be substantiated by our experiments nor with data of another recent clinical-prospective study [8]. Moreover, our findings of significantly increased flow rates at higher pressures were in line with previous clinical studies [7,8]. The pressure-dependent differences in the flow rates may likely be explained by the intraosseous pressure of approximately 20 to 30 mmHg (one third of the systemic mean pressure)[22], which is partly neutralizing the infusion pressure.

The correlations between the pressure levels and the infused volumes indicate that applying external pressure allows some control over the volume infused (Fig 2). However, the cumulative volume and flow rates were accompanied by large standard deviations and consequently by large variation coefficients, indicating that the flow rates of each device are highly individual (Tables 2 and 3). In the EZ-IO tibia and humerus, flow rate variations tended to be smaller than with the FASTR. As a consequence, in an emergency situation, the flow rates might be much lower than one may expect on the basis of the mean values. Therefore, our results cannot confirm the findings of Pasley et al. [20], stating that the flow rates of sternal puncture are highly consistent in a 5-minute timeframe. To our surprise, no correlations of the flow rates could be found with the donors’ morphometrics, further indicating that achievable flow rates might be hard to predict. Depending on the infusion characteristics and clinical scenarios, it will probably be necessary to augment the infusion rates of intraosseous lines with a pressure bag or rapid infusion device.

Flow rates decreased in the FASTR and the EZ-IO tibia at increasing durations irrespective of the external pressure applied. This was true for the unfixed and fixed donors (Tables 2 and 3). In contrast, the flow rates tended to slightly increase using the EZ-IO humerus. Apart from the device characteristics, another explanation for the site- and time-dependent flow rates may be found in the morphology of the respective bones and their vascular drainage. Both the humerus and the tibia have a well-defined bone cavity and nutrient vessels in their meta- or proximal diaphyses [23,24], namely the posterior and aberrant anterior tibial vessels at the tibia [25], the circumflex vessels and nutrient branches originating from the deep brachial artery for the humerus [26]. Immediately after intraosseous puncture and following an initial 10-ml flush, higher flow rates may be explained by filling of the bone cavities. Once this mechanism ends, additional volume gain can only be achieved through the bones’ vascular drainage defining the flow characteristics and explaining the drop in the flow rates. In contrast, sternal bone cavities are much smaller and flatter. A few vessels directly give branches to the sternum, namely the internal thoracic artery with the anterior intercostal rami or mediastinal branches [27–33]. Consequently, the sternal bone cavity may fill faster, explaining the pronounced time-dependent drop in the flow rates. Yet, given the large number of sternal vessels, drainage appears to be more effective to the end that higher flow rates can be maintained than in the humerus or tibia. Also, beyond pressure levels of systemic blood pressure, the arteries may participate in draining the volume from the respective bones.

The EZ-IO tibia was rated the highest by both the technically inexperienced participants and the observers and accompanied by the highest first-pass success, least insertion times and lowest complication rates. Though the participants gave the FASTR an equal rating as the EZ-IO tibia, observer rating was the lowest for the FASTR compared to the EZ-IO tibia and humerus. Moreover, first-pass success was lower with the FASTR, insertion times were longer and device-related complications more frequent. The participants also rated the EZ-IO humerus lower than the EZ-IO tibia. The FASTR and the EZ-IO humerus were accompanied by the most donor-related complications. Levitan et al. [34] observed similar findings with first-pass success rates higher than 95% in medical providers following a 5-minute introduction. Our findings were in line with the results from Brenner et al. [13] who found first-pass success rates of 98% for the EZ-IO tibia in body donors in comparison to the first-pass success rates of 80% using a manual needle technique. These reproducible data indicate that the EZ-IO device is easy to use and requires minimal training. Lower success rates of 82% were described by Kurowski et al. [35] in 107 paramedics applying for the EZ-IO system in phantoms. Furthermore, in their study, the participants evaluated the EZ-IO better than the Jamshidi needle but worse than the bone injection gun, which might partly be related due to safety issues [35].

Safety issues are important to consider in this setting along with the lack of reliability in the flow rates due to the large variations especially with the FASTR, but also with the EZ-IO. A number of participants were doubtful when using the EZ-IO humerus correctly to exclude brachial artery and plexus or axillar nerve injury. The same was true for the FASTR due to the proximity to the heart and the potential existence of sternal foramina [36–38]. Another issue is the limitation to a single attempt with the FASTR, whereas two humeri or tibia were allowed for another try in intraosseous puncture. Apparently, in the case of first-time users, the technical ease of the device and the anatomical safety of the puncture site outweigh the flow rates.

### Limitations

First, only a limited number of devices, participants and body donors were available for the given study, which is reflected by the statistical power of 0.62. Therefore there remains some uncertainty regarding our null finding, which may nonetheless fail to exclude a clinically important effect. However, given the costs related to the equipment and the body donation as well as limitations in the availability of body donors it was impossible to include larger numbers with the given setup. Future studies may help substantiate our findings with larger sample sizes to overcome this weakness of our study. Second, ethanol-fixed donors were used for safety reasons with the technically inexperienced participants instead of unfixed donors. Though visual and haptic properties remained close to the unfixed condition [15,16], the fixation lowered the flow rates of the intraosseous devices. This might have partially been related to altered flow characteristics due to the fixatives, thrombosed bone marrow and vessels. To overcome this issue, we determined flow rates in unfixed donors and used these data to normalize the values of the fixed ones, in line with previous studies on intraosseous devices in patients [7,8] as well as in fixed body donors [20]. Being well aware that these normalizing values are estimation, the use of fixed body donors remains a limitation of the study in addition to post-mortem changes of the vascular system.

### Conclusions

The EZ-IO and FASTR devices may be effective for fluid resuscitation and drug application even by first-time applicants. Variations in the flow rates may limit their reliability. Though the highest flow rates can be achieved with the FASTR, small variations in the flow rates, excellent user rating, the shortest insertion times and the least complications clearly speak in favor of the EZ-IO tibia.

### Key messages

1. The first-pass insertion success rates of the FASTR and the EZ-IO tibia were comparable and significantly higher than with the EZ-IO humerus.
2. Flow rates were pressure-dependent but varied highly among the devices.
3. Flow rates and cumulative volumes were larger using the FASTR, compared to the EZ-IO tibia and humerus, but without differing significantly.
4. The EZ-IO and FASTR devices appear to be effective for fluid resuscitation and drug application even by first-time applicants.

## Supporting Information

S1 Table Ethanol-fixed donors and unfixed donors were similar in age (85.8±6.5 vs. 87.0±2.0 years), gender (17♀/10♂ vs. 1♀/2♂), body weight (68.3±13.2 vs. 74.3±11.6 kg) and BMI (26.0±5.5 vs. 24.6±3.9 kg/m^2^) were similar and non-significantly different with the only exception of body length (162.5±7.6 vs. 174.0±5.3 cm). (DOCX)

S2 Table Participant characteristics. Participant age (22.1±3.0 vs. 23.9±5.8 vs. 21.8±1.2 years), semester (4.6±1.5 vs. 4.0±0.0 vs. 4.4±1.3 semesters) and gender (3♀/4♂ vs. 3♀/7♂ vs. 5♀/5♂) were similar and non-significantly different for the three groups.
(DOCX)

S3 Table Detailed data on participant grading, insertion time, number of attempts and complications. (DOCX)

S4 Table Pressure-dependent cumulative volumes in unfixed donors. (DOCX)

S5 Table Pressure-dependent flow rates in unfixed donors. (DOCX)

S6 Table Flow rates in ethanol-fixed body donors (not normalized). (DOCX)

S7 Table Cumulative volumes in ethanol-fixed body donors (not normalized). (DOCX)
