# Supplementary material for: Comparison of the Fluid Resuscitation Rate with and without External Pressure Using Two Intraosseous Infusion Systems for Adult Emergencies, the CITRIN (Comparison of InTRaosseous infusion systems in emergency medicINe)-Study
Source: PLoS One. 2015 Dec 2;10(12):e0143726. doi: 10.1371/journal.pone.0143726 (PMC4668027; doi:10.1371/journal.pone.0143726)
Supplement: S1 Table — (DOCX) [file pone.0143726.s001.docx]

**S1 Table: Ethanol-fixed donors and unfixed donors were similar in age (85.8±6.5 vs. 87.0±2.0 years), gender (17♀/10♂ vs. 1♀/2♂), body weight (68.3±13.2 vs. 74.3±11.6 kg) and BMI (26.0±5.5 vs. 24.6±3.9 kg/m^2^) were similar and non-significantly different with the only exception of body length (162.5±7.6 vs. 174.0±5.3 cm).**

| **Specimen** | **Age** | **Body** | | **BMI** | **Gender** | **Cause of death** |
| --- | --- | --- | --- | --- | --- | --- |
|  |  | **weight [kg]** | **length [cm]** |  |  |  |
|  |  |  |  |  |  |  |
| **Ethanol-fixed** |  |  |  |  |  |  |
| 1-14 | 84 | 59 | 171 | 20 | ♀ | Stroke |
| 2-14 | 85 | 68 | 164 | 25 | ♂ | Cardiac insufficiency |
| 3-14 | 86 | 81 | 161 | 31 | ♂ | Pneumonia |
| 4-14 | 92 | 63 | 150 | 28 | ♀ | Cardiac shock |
| 5-14 | 92 | 78 | 159 | 31 | ♀ | Cardiac shock |
| 6-14 | 87 | 74 | 168 | 26 | ♂ | Stroke |
| 7-14 | 87 | 74 | 151 | 32 | ♀ | Acute cadiac infarction |
| 9-14 | 72 | 58 | 153 | 25 | ♀ | Cerebrovascular insult |
| 11-14 | 78 | 78 | 164 | 29 | ♂ | Cardiac insufficiency |
| 12-14 | 84 | 52 | 151 | 23 | ♀ | Cardiac insufficiency |
| 13-14 | 83 | 54 | 151 | 24 | ♀ | Stroke |
| 14-14 | 88 | 48 | 167 | 17 | ♀ | Acute cadiac infarction |
| 15-14 | 90 | 59 | 158 | 24 | ♀ | Endocarditis |
| 16-14 | 89 | 60 | 175 | 20 | ♂ | Cardiac insufficiency |
| 18-14 | 91 | 72 | 165 | 26 | ♀ | Respiratory insufficiency |
| 19-14 | 79 | 56 | 165 | 21 | ♀ | Chronic obstructive pulmonary disease |
| 20-14 | 89 | 76 | 168 | 27 | ♂ | Cardiac insufficiency |
| 24-14 | 90 | 58 | 166 | 21 | ♀ | Stroke |
| 25-14 | 79 | 73 | 176 | 24 | ♂ | Septic shock |
| 26-14 | 95 | 89 | 157 | 36 | ♀ | Cardiac insufficiency |
| 27-14 | 69 | 50 | 168 | 18 | ♀ | Bronchial carcionoma |
| 28-14 | 86 | 98 | 160 | 38 | ♂ | Acute cadiac infarction |
| 29-14 | 94 | 76 | 161 | 29 | ♂ | unclear |
| 35-14 | 90 | 84 | 170 | 29 | ♀ | Respiratory insufficiency |
|  |  |  |  |  |  |  |
| **Mean value** | **85.8** | **68.3** | **162.5** | **26.0** |  |  |
| **Standard deviation** | **6.5** | **13.1** | **7.6** | **5.5** |  |  |
|  |  |  |  |  |  |  |
| **Fresh** |  |  |  |  |  |  |
| 43-15 | 87 | 86 | 172 | 29 | ♀ | Cardiac insufficiency |
| 44-15 | 89 | 73 | 180 | 23 | ♂ | Acute cadiac infarction |
| 46-15 | 85 | 64 | 170 | 22 | ♂ | Femoral fracturing |
|  |  |  |  |  |  |  |
| **Mean value** | **87.0** | **74.3** | **174.0** | **24.6** |  |  |
| **Standard deviation** | **2.0** | **11.1** | **5.3** | **3.9** |  |  |
| ***p value fresh vs. fixed*** | ***0.756*** | ***0.452*** | ***0.018*** | ***0.680*** | ***0.351*** |  |
