# Supplementary material for: Comparison of the Fluid Resuscitation Rate with and without External Pressure Using Two Intraosseous Infusion Systems for Adult Emergencies, the CITRIN (Comparison of InTRaosseous infusion systems in emergency medicINe)-Study
Source: PLoS One. 2015 Dec 2;10(12):e0143726. doi: 10.1371/journal.pone.0143726 (PMC4668027; doi:10.1371/journal.pone.0143726)
Supplement: S2 Table — Participant age (22.1±3.0 vs. 23.9±5.8 vs. 21.8±1.2 years), semester (4.6±1.5 vs. 4.0±0.0 vs. 4.4±1.3 semesters) and gender (3♀/4♂ vs. 3♀/7♂ vs. 5♀/5♂) were similar and non-significantly different for the three groups. (DOCX) [file pone.0143726.s002.docx]

**S2 Table: Participant characteristics.** Participant age (22.1±3.0 vs. 23.9±5.8 vs. 21.8±1.2 years), semester (4.6±1.5 vs. 4.0±0.0 vs. 4.4±1.3 semesters) and gender (3♀/4♂ vs. 3♀/7♂ vs. 5♀/5♂) were similar and non-significantly different for the three groups.

|  | **Gender** | **Age** | **Semester** | **Donor and devices** | |
| --- | --- | --- | --- | --- | --- |
| **Group 1** |  |  |  |  |  |
| 1 | ♂ | 19 | 4 | - | 4 |
| 2 | ♀ | 20 | 4 | - | 5 |
| 3 | ♀ | 21 | 4 | - | 9 |
| 4 | ♀ | 21 | 4 | - | 7 |
| 5 | ♂ | 22 | 4 | - | 6 |
| 6 | ♂ | 24 | 8 | 1 | 1 |
| 7 | ♂ | 28 | 4 | 2 | 2 |
| **Mean value** | **3 ♀ / 4 ♂** | **22.1** | **4.6** |  |  |
| **Standard deviation** |  | **3.0** | **1.5** |  |  |
|  |  |  |  |  |  |
| **Group 2** |  |  |  |  |  |
| 8 | ♂ | 20 | 4 | 4 | 20 |
| 9 | ♀ | 20 | 4 | 14 | 29 |
| 10 | ♀ | 20 | 4 | 18 | - |
| 11 | ♂ | 21 | 4 | 20 | 15 |
| 12 | ♂ | 21 | 4 | 26 | - |
| 13 | ♀ | 22 | 4 | 12 | 12 |
| 14 | ♂ | 24 | 4 | 24 | 16 |
| 15 | ♂ | 25 | 4 | 28 | 24 |
| 16 | ♂ | 27 | 4 | 6 | 14 |
| 17 | ♂ | 39 | 4 | 16 | 35 |
| **Mean value** | **3 ♀ / 7 ♂** | **23.9** | **4.0** |  |  |
| **Standard deviation** |  | **5.8** | **0.0** |  |  |
|  |  |  |  |  |  |
| **Group 3** |  |  |  |  |  |
| 18 | ♀ | 20 | 4 | 3 | - |
| 19 | ♀ | 20 | 4 | 19 | - |
| 20 | ♂ | 21 | 4 | 27 | - |
| 21 | ♂ | 22 | 4 | 7 | - |
| 22 | ♂ | 22 | 4 | 25 | - |
| 23 | ♀ | 23 | 4 | 15 | - |
| 24 | ♀ | 19 | 4 | 13 | 18 |
| 25 | ♂ | 20 | 4 | 9 | 11 |
| 26 | ♂ | 24 | 8 | 11 | 11 |
| 27 | ♀ | 27 | 4 | 5 | 19 |
| **Mean value** | **5 ♀ / 5 ♂** | **21.8** | **4.4** |  |  |
| **Standard deviation** |  | **2.39** | **1.26** |  |  |
|  |  |  |  |  |  |
| **Overall participants** |  |  |  |  |  |
|  |  |  |  |  |  |
| **Mean value** | **11 ♀ / 16 ♂** | **22.7** | **4.3** |  |  |
| **Standard deviation** |  | **4.1** | **1.1** |  |  |
| ***p value inter group*** | ***0.683*** | ***0.497*** | ***0.746*** |  |  |
