# Supplementary material for: Comparison of the Fluid Resuscitation Rate with and without External Pressure Using Two Intraosseous Infusion Systems for Adult Emergencies, the CITRIN (Comparison of InTRaosseous infusion systems in emergency medicINe)-Study
Source: PLoS One. 2015 Dec 2;10(12):e0143726. doi: 10.1371/journal.pone.0143726 (PMC4668027; doi:10.1371/journal.pone.0143726)
Supplement: S3 Table — (DOCX) [file pone.0143726.s003.docx]

**S3 Table: Detailed data on participant grading, insertion time, number of attempts and complications.**

| **Participants** | **Grading** | | | | | |  | **Insertion time** |  |  | **Attempt** |  | **Complications** | | | | | |
| --- | --- | --- | --- | --- | --- | --- | --- | --- | --- | --- | --- | --- | --- | --- | --- | --- | --- | --- |
| **[number]** | **[1 = excellent to 6 = failure]** | | | | | |  | **[sec]** |  |  | **[number]** |  |  |  |  |  |  |  |
|  | **Participant** | | | **Observer** | | |  |  |  |  |  |  | **donor** | **device** | **donor** | **device** | **donor** | **device** |
|  | **EZ-IO tibia** | **EZ-IO humerus** | **FASTR** | **EZ-IO Tibia** | **EZ-IO humerus** | **FASTR** | **EZ-IO tibia** | **EZ-IO humerus** | **FASTR** | **EZ-IO tibia** | **EZ-IO humerus** | **FASTR** | **EZ-IO tibia** | | **EZ-IO humerus** | | **FASTR** | |
| 1 |  |  | 2 |  |  | 1 |  |  | 30 |  |  | 1 |  |  |  |  | flow |  |
| 2 |  |  | 3 |  |  | 1 |  |  | 110 |  |  | 1 |  |  |  |  |  |  |
| 3 |  |  | 1 |  |  | 1 |  |  | 45 |  |  | 1 |  |  |  |  |  | securing |
| 4 |  |  | 1 |  |  | 1 |  |  | 28 |  |  | 1 |  |  |  |  |  |  |
| 5 |  |  | 3 |  |  | 1 |  |  | 33 |  |  | 1 |  |  |  |  |  |  |
| 6 | 1 | 1 | 1 | 1 | 1 | 1 | 19 | 5 | 17 | 1 | 1 | 1 |  |  |  |  |  |  |
| 7 | 2 | 2 | 3 | 2 | 2 | 2 | 28 | 11.5 | 14 | 2 | 1 | 1 |  |  |  |  | flow | securing |
| 8 | 1 | 3 | 1 | 1 | 2 | 1 | 15 | 210 | 23 | 1 | 2 | 1 |  |  | flow |  |  |  |
| 9 | 2 | 1 | 3 | 1 | 1 | 1 | 11 | 12 | 45 | 1 | 1 | 2 |  |  |  |  |  |  |
| 10 | 2 | 3 |  | 1 | 2 |  | 14 | 38 |  | 1 | 2 |  |  |  |  |  |  |  |
| 11 | 2 | 3 | 2 | 1 | 2 | 1 | 22 | 20 | 24 | 1 | 2 | 1 |  |  |  |  |  |  |
| 12 | 1 | 2 |  | 1 | 1 |  | 23 | 43 |  | 1 | 1 |  |  |  |  |  |  | securing |
| 13 | 1 | 1 | 1 | 1 | 1 | 1 | 10 | 20 | 25 | 1 | 2 | 1 |  |  | flow |  |  |  |
| 14 | 1 | 2 | 1 | 1 | 1 | 1 | 10 | 20 | 35 | 1 | 1 | 1 |  |  |  |  |  |  |
| 15 | 2 | 3 | 1 | 1 | 3 | 1 | 12 | 36 | 32 | 1 | 1 | 1 |  |  |  |  |  |  |
| 16 | 1 | 3 | 2 | 1 | 1 | 1 | 33 | 30 | 28 | 1 | 1 | 1 |  |  | flow |  |  |  |
| 17 | 1 | 3 | 1 | 1 | 1 | 1 | 20 | 18 | 14 | 1 | 1 | 1 |  |  |  |  |  |  |
| 18 | 2 | 2 |  | 1 | 2 |  | 23 | 54 |  | 1 | 1 |  |  |  |  |  |  |  |
| 19 | 1 | 1 |  | 1 | 1 |  | 10 | 15 |  | 1 | 1 |  |  |  |  |  |  |  |
| 20 | 1 | 2 |  | 1 | 1 |  | 14 | 20 |  | 1 | 1 |  |  |  |  |  |  |  |
| 21 | 1 | 3 |  | 1 | 1 |  | 30 | 20 |  | 1 | 2 |  |  |  | flow |  |  |  |
| 22 | 1 | 2 |  | 1 | 1 |  | 13 | 9 |  | 1 | 1 |  |  |  |  |  |  |  |
| 23 | 1 | 1 |  | 1 | 1 |  | 13 | 6 |  | 1 | 1 |  |  |  |  |  |  |  |
| 24 | 1 | 1 | 1 | 1 | 2 | 1 | 15 | 10 | 32 | 1 | 1 | 1 |  |  |  |  |  |  |
| 25 | 1 | 2 | 1 | 1 | 1 | 1 | 10 | 15 | 32 | 1 | 1 | 1 |  | needle |  |  |  |  |
| 26 | 1 | 2 | 1 | 1 | 1 | 1 | 8 | 12 | 20 | 1 | 1 | 1 |  |  |  |  |  |  |
| 27 | 1 | 1 | 2 | 1 | 1 | 1 | 22 | 15 | 32 | 2 | 1 | 1 | flow |  | flow |  |  |  |
| **Mean value** | **1.3** | **2.0** | **1.6** | **1.0** | **1.4** | **1.1** | **17.0** | **29.1** | **32.6** | **1.1** | **1.2** | **1.1** |  |  |  |  |  |  |
| **Standard deviation** | **0.5** | **0.8** | **0.8** | **0.2** | **0.6** | **0.2** | **7.2** | **42.3** | **20.6** | **0.3** | **0.4** | **0.2** |  |  |  |  |  |  |
| **Median** | **1** | **2** | **1** | **1** | **1** | **1** | **15** | **19** | **30** | **1** | **1** | **1** |  |  |  |  |  |  |
| **Min** | **1** | **1** | **1** | **1** | **1** | **1** | **8** | **5** | **14** | **1** | **1** | **1** |  |  |  |  |  |  |
| **Max** | **2** | **3** | **3** | **2** | **3** | **2** | **33** | **210** | **110** | **2** | **2** | **2** |  |  |  |  |  |  |
| ***P value (ANOVA)*** | ***0.008*** | | | ***0.012*** | | | ***0.165*** | | | ***0.209*** | | |  |  |  |  |  |  |
| ***Post-hoc analysis (if applicable)*** |  |  |  |  |  |  |  |  |  |  |  |  |  |  |  |  |  |  |
| *EZ-IO tibia vs. EZ-IO humerus* |  | *0.006* |  |  | *0.025* |  |  |  |  |  |  |  |  |  |  |  |  |  |
| *EZ-IO humerus vs. FASTR* |  | *0.390* |  |  | *0.039* |  |  |  |  |  |  |  |  |  |  |  |  |  |
| *EZ-IO tibia vs. EZ-IO humerus* |  | *0.349* |  |  | *1.0* |  |  |  |  |  |  |  |  |  |  |  |  |  |
