# Supplementary material for: Comparison of the Fluid Resuscitation Rate with and without External Pressure Using Two Intraosseous Infusion Systems for Adult Emergencies, the CITRIN (Comparison of InTRaosseous infusion systems in emergency medicINe)-Study
Source: PLoS One. 2015 Dec 2;10(12):e0143726. doi: 10.1371/journal.pone.0143726 (PMC4668027; doi:10.1371/journal.pone.0143726)
Supplement: S4 Table — (DOCX) [file pone.0143726.s004.docx]

**S4 Table: Pressure-dependent cumulative volumes in unfixed donors.**

| **Pressure** | | **EZ-IO tibia [ml] at min** | | |  | **EZ-IO humerus [ml] at min** | | |  | **FASTR [ml] at min** | | |
| --- | --- | --- | --- | --- | --- | --- | --- | --- | --- | --- | --- | --- |
| **[mmHg]** | | **1** | **3** | **5** |  | **1** | **3** | **5** |  | **1** | **3** | **5** |
|  |  |  |  |  |  |  |  |  |  |  |  |  |
| **0** | **Mean value** | 26.9 | 62.0 | 103.5 |  | 15.9 | 48.1 | 79.6 |  | 53.2 | 120.3 | 188.5 |
|  | **Standard deviation** | 5.0 | 26.0 | 54.5 |  | 3.4 | 21.0 | 42.6 |  | 2.0 | 13.3 | 35.5 |
| **50** | **Mean value** | 34.5 | 94.1 | 144.8 |  | 29.8 | 76.3 | 125.3 |  | 54.5 | 136.3 | 215.2 |
|  | **Standard deviation** | 25.1 | 69.5 | 101.9 |  | 22.2 | 36.9 | 69.9 |  | 0.8 | 27.1 | 58.6 |
| **100** | **Mean value** | 48.7 | 131.6 | 210.1 |  | 29.2 | 89.9 | 138.6 |  | 46.2 | 144.8 | 237.3 |
|  | **Standard deviation** | 30.7 | 80.7 | 137.1 |  | 17.6 | 48.3 | 80.7 |  | 16.9 | 66.2 | 94.8 |
| **150** | **Mean value** | 52.7 | 130.5 | 226.7 |  | 29.2 | 96.5 | 176.8 |  | 74.9 | 186.7 | 301.6 |
|  | **Standard deviation** | 47.9 | 99.7 | 148.5 |  | 21.1 | 58.0 | 72.4 |  | 34.8 | 88.8 | 146.2 |
| **200** | **Mean value** | 63.9 | 177.7 | 277.1 |  | 34.5 | 116.1 | 190.5 |  | 81.2 | 208.5 | 348.2 |
|  | **Standard deviation** | 50.1 | 108.6 | 175.2 |  | 21.6 | 43.5 | 59.8 |  | 30.9 | 56.2 | 94.9 |
| **250** | **Mean value** | 59.3 | 148.7 | 273.7 |  | 46.1 | 99.8 | 198.3 |  | 98.8 | 261.5 | 427.5 |
|  | **Standard deviation** | 52.4 | 143.2 | 195.2 |  | 34.7 | 60.3 | 99.3 |  | 33.2 | 104.7 | 153.2 |
| **300** | **Mean value** | 69.2 | 205.7 | 331.4 |  | 60.6 | 169.4 | 247.7 |  | 111.8 | 282.5 | 459.5 |
|  | **Standard deviation** | 54.1 | 171.0 | 246.6 |  | 44.4 | 109.8 | 129.4 |  | 47.1 | 118.6 | 184.4 |
