# Supplementary material for: Comparison of the Fluid Resuscitation Rate with and without External Pressure Using Two Intraosseous Infusion Systems for Adult Emergencies, the CITRIN (Comparison of InTRaosseous infusion systems in emergency medicINe)-Study
Source: PLoS One. 2015 Dec 2;10(12):e0143726. doi: 10.1371/journal.pone.0143726 (PMC4668027; doi:10.1371/journal.pone.0143726)
Supplement: S5 Table — (DOCX) [file pone.0143726.s005.docx]

**S5 Table: Pressure-dependent flow rates in unfixed donors.**

| **Pressure** | | **EZ-IO tibia [ml/min] at min** | | |  | **EZ-IO humerus [ml/min] at min** | | |  | **FASTR [ml/min] at min** | | |
| --- | --- | --- | --- | --- | --- | --- | --- | --- | --- | --- | --- | --- |
| **[mmHg]** | | **1** | **3** | **5** |  | **1** | **3** | **5** |  | **1** | **3** | **5** |
|  |  |  |  |  |  |  |  |  |  |  |  |  |
| **0** | **Mean value** | 26.9 | 20.7 | 20.7 |  | 15.9 | 16.0 | 15.9 |  | 53.2 | 40.1 | 37.7 |
|  | **Standard deviation** | 5.0 | 8.7 | 10.9 |  | 3.4 | 7.0 | 8.5 |  | 2.0 | 4.4 | 7.1 |
| **50** | **Mean value** | 34.5 | 31.4 | 29.0 |  | 29.8 | 25.4 | 25.1 |  | 54.5 | 45.4 | 43.0 |
|  | **Standard deviation** | 25.1 | 23.2 | 20.4 |  | 22.2 | 12.3 | 14.0 |  | 0.8 | 9.0 | 11.7 |
| **100** | **Mean value** | 48.7 | 43.9 | 42.0 |  | 29.2 | 30.0 | 27.7 |  | 46.2 | 48.3 | 47.5 |
|  | **Standard deviation** | 30.7 | 26.9 | 27.4 |  | 17.6 | 16.1 | 16.1 |  | 16.9 | 22.1 | 19.0 |
| **150** | **Mean value** | 52.7 | 43.5 | 45.3 |  | 29.2 | 32.2 | 35.4 |  | 74.9 | 62.2 | 60.3 |
|  | **Standard deviation** | 47.9 | 33.2 | 29.7 |  | 21.1 | 19.3 | 14.5 |  | 34.8 | 29.6 | 29.2 |
| **200** | **Mean value** | 63.9 | 59.2 | 55.4 |  | 34.5 | 38.7 | 38.1 |  | 81.2 | 69.5 | 69.6 |
|  | **Standard deviation** | 50.1 | 36.2 | 35.0 |  | 21.6 | 14.5 | 12.0 |  | 30.9 | 18.7 | 19.0 |
| **250** | **Mean value** | 59.3 | 49.6 | 54.7 |  | 46.1 | 33.3 | 39.7 |  | 98.8 | 87.2 | 85.5 |
|  | **Standard deviation** | 52.4 | 47.7 | 39.0 |  | 34.7 | 20.1 | 19.9 |  | 33.2 | 34.9 | 30.6 |
| **300** | **Mean value** | 69.2 | 68.6 | 66.3 |  | 60.6 | 56.5 | 49.5 |  | 111.8 | 94.2 | 91.9 |
|  | **Standard deviation** | 54.1 | 57.0 | 49.3 |  | 44.4 | 36.6 | 25.9 |  | 47.1 | 39.5 | 36.9 |
|  | ***Correlation***  ***(pressure and flow rate)*** | ***0.962***** | ***0.950***** | ***0.982***** |  | ***0.937***** | ***0.932***** | ***0.987***** |  | ***0.932***** | ***0.979***** | ***0.986***** |
|  |  |  |  |  |  |  |  |  |  |  |  |  |
