# Supplementary material for: Comparison of the Fluid Resuscitation Rate with and without External Pressure Using Two Intraosseous Infusion Systems for Adult Emergencies, the CITRIN (Comparison of InTRaosseous infusion systems in emergency medicINe)-Study
Source: PLoS One. 2015 Dec 2;10(12):e0143726. doi: 10.1371/journal.pone.0143726 (PMC4668027; doi:10.1371/journal.pone.0143726)
Supplement: S6 Table — (DOCX) [file pone.0143726.s006.docx]

**S6 Table: Flow rates in ethanol-fixed body donors (not normalized).**

| **Number** | **EZ-IO Tibia** | | | | | |  | **EZ-IO Humerus** | | | | | |  | **FASTR** | | | | | |
| --- | --- | --- | --- | --- | --- | --- | --- | --- | --- | --- | --- | --- | --- | --- | --- | --- | --- | --- | --- | --- |
|  | **0 mmHg** | | | **150 mmHg** | | |  | **0 mmHg** | | | **150 mmHg** | | |  | **0 mmHg** | | | **150 mmHg** | | |
|  | **1 min** | **3 min** | **5 min** | **1 min** | **3 min** | **5 min** |  | **1 min** | **3 min** | **5 min** | **1 min** | **3 min** | **5 min** |  | **1 min** | **3 min** | **5 min** | **1 min** | **3 min** | **5 min** |
|  |  |  |  |  |  |  |  |  |  |  |  |  |  |  |  |  |  |  |  |  |
| 1 |  |  |  |  |  |  |  |  |  |  |  |  |  |  | 17.8 | 23.8 | 28.7 | 11.9 | 23.8 | 45.6 |
| 2 |  |  |  |  |  |  |  |  |  |  |  |  |  |  | 22.8 | 70.4 | 96.1 | 85.2 | 150.6 | 129.8 |
| 3 |  |  |  |  |  |  |  |  |  |  |  |  |  |  | 10.9 | 14.9 | 15.8 | 1.0 | 8.9 | 14.9 |
| 4 |  |  |  |  |  |  |  |  |  |  |  |  |  |  |  |  |  |  |  |  |
| 5 |  |  |  |  |  |  |  |  |  |  |  |  |  |  | 5.9 | 10.9 | 14.9 | 14.9 | 43.6 | 70.3 |
| 6 | 6.9 | 19.8 | 34.7 | 38.6 | 61.4 | 92.2 |  | 2.0 | 4.0 | 5.0 | 5.0 | 15.9 | 29.7 |  | 11.9 | 25.8 | 40.6 | 14.9 | 74.3 | 109.0 |
| 7 | 1.0 | 4.0 | 5.9 | 26.8 | 50.5 | 81.3 |  | 1.0 | 3.0 | 3.0 | 3.0 | 19.8 | 39.6 |  |  |  |  |  |  |  |
| 8 | 2.0 | 8.9 | 8.9 | 0.0 | 5.0 | 5.9 |  | 5.0 | 11.9 | 18.8 | 4.0 | 8.9 | 23.8 |  | 0.0 | 3.0 | 4.0 | 13.9 | 21.8 | 26.7 |
| 9 | 12.9 | 19.8 | 23.8 | 3.0 | 16.8 | 27.7 |  | 5.0 | 11.9 | 19.8 | 5.0 | 22.8 | 28.7 |  | 10.9 | 26.7 | 45.6 | 18.8 | 95.1 | 156.5 |
| 10 | 7.9 | 18.8 | 31.7 | 23.8 | 47.6 | 75.3 |  | 14.9 | 31.7 | 52.5 | 25.8 | 43.6 | 89.2 |  |  |  |  |  |  |  |
| 11 | 3.0 | 14.9 | 31.7 | 18.8 | 46.6 | 76.3 |  | 4.0 | 11.9 | 21.8 | 5.0 | 23.8 | 37.7 |  | 1.0 | 3.0 | 5.0 | 1.0 | 27.0 | 12.0 |
| 12 | 5.0 | 17.8 | 30.7 | 21.8 | 66.4 | 112.0 |  | 2.0 | 2.0 | 3.0 | 17.8 | 20.8 | 23.8 |  |  |  |  |  |  |  |
| 13 | 1.0 | 1.0 | 4.0 | 5.9 | 8.9 | 10.9 |  | 11.9 | 26.8 | 45.6 | 68.4 | 118.9 | 180.3 |  | 2.0 | 4.0 | 10.9 | 5.0 | 6.9 | 39.6 |
| 14 | 0.0 | 3.0 | 6.9 | 9.9 | 27.7 | 43.6 |  | 2.0 | 3.0 | 5.9 | 2.0 | 7.9 | 12.9 |  | 14.9 | 31.7 | 54.5 | 6.9 | 40.6 | 56.5 |
| 15 | 5.0 | 6.9 | 7.9 | 9.9 | 11.9 | 20.8 |  | 4.0 | 6.9 | 13.9 | 5.9 | 24.8 | 41.6 |  | 0.0 | 1.0 | 2.0 | 0.0 | 3.0 | 5.9 |
| 16 | 9.9 | 17.8 | 19.8 | 6.9 | 10.9 | 25.8 |  | 0.0 | 0.0 | 0.0 | 8.9 | 9.9 | 14.9 |  | 7.9 | 17.8 | 29.7 | 19.8 | 42.6 | 59.5 |
| 17 | 13.9 | 17.8 | 17.8 | 12.9 | 25.8 | 31.7 |  | 2.0 | 4.0 | 8.9 | 7.9 | 17.8 | 37.7 |  | 1.0 | 2.0 | 4.0 | 4.0 | 8.9 | 12.9 |
| 18 | 7.9 | 20.8 | 23.8 | 3.0 | 12.9 | 27.7 |  | 26.8 | 51.5 | 56.5 | 18.8 | 22.8 | 28.7 |  |  |  |  |  |  |  |
| 19 | 10.9 | 43.6 | 48.6 | 5.0 | 8.9 | 17.8 |  | 19.8 | 24.8 | 32.7 | 51.5 | 52.5 | 54.5 |  |  |  |  |  |  |  |
| 20 | 1.0 | 4.0 | 5.0 | 20.8 | 30.7 | 37.7 |  | 28.7 | 30.7 | 33.7 | 2.0 | 5.0 | 11.9 |  |  |  |  |  |  |  |
| 21 | 1.0 | 3.0 | 5.0 | 33.7 | 62.4 | 82.2 |  | 2.0 | 3.0 | 4.0 | 13.9 | 21.8 | 28.7 |  |  |  |  |  |  |  |
| 22 | 1.0 | 4.0 | 5.0 | 5.0 | 12.9 | 17.8 |  | 5.9 | 9.9 | 15.9 | 5.9 | 28.7 | 39.6 |  |  |  |  |  |  |  |
| 23 | 2.0 | 2.0 | 4.0 | 0.0 | 2.0 | 3.0 |  | 4.0 | 8.9 | 24.8 | 26.8 | 31.7 | 35.7 |  |  |  |  |  |  |  |
| 24 | 0.0 | 4.0 | 5.0 | 3.0 | 5.9 | 6.9 |  | 2.0 | 6.9 | 8.9 | 6.9 | 10.9 | 21.8 |  | 4.0 | 13.9 | 22.8 | 14.0 | 41.0 | 30.0 |
| 25 | 8.9 | 15.9 | 17.8 | 5.9 | 21.8 | 30.7 |  | 5.9 | 8.9 | 13.9 | 37.7 | 90.2 | 99.1 |  | 10.9 | 18.8 | 26.7 | 56.5 | 119.8 | 162.4 |
| 26 | 5.9 | 8.9 | 20.8 | 5.0 | 41.6 | 67.4 |  | 12.9 | 14.9 | 24.8 | 33.7 | 51.5 | 64.4 |  | 2.0 | 5.9 | 6.9 | 5.0 | 6.9 | 39.6 |
| 27 | 9.9 | 17.8 | 19.8 | 13.9 | 16.8 | 23.8 |  | 0.0 | 6.9 | 8.9 | 11.9 | 17.8 | 23.8 |  | 2.0 | 5.0 | 6.9 | 10.9 | 27.8 | 36.7 |
|  |  |  |  |  |  |  |  |  |  |  |  |  |  |  |  |  |  |  |  |  |
| **Mean value** | **5.3** | **12.5** | **17.2** | **12.4** | **27.1** | **41.8** |  | **7.3** | **12.9** | **19.2** | **16.7** | **30.4** | **44.0** |  | **7.4** | **16.4** | **24.4** | **16.7** | **43.7** | **59.3** |
| **Standard deviation** | **4.4** | **10.0** | **12.6** | **11.0** | **20.6** | **31.9** |  | **8.4** | **12.7** | **16.3** | **17.6** | **27.5** | **37.7** |  | **6.9** | **17.0** | **24.4** | **21.9** | **42.6** | **50.2** |
| ***p value inner group*** | ***0.001 - 0.013*** | | | | | |  | ***0.000 - 0.010*** | | | | | |  | ***0.002 - 0.054*** | | | | | |
| ***p value inter group*** | ***0.447 - 0.616*** | | | | | | | | | | | | | | | | | | | |
