# Supplementary material for: Comparison of the Fluid Resuscitation Rate with and without External Pressure Using Two Intraosseous Infusion Systems for Adult Emergencies, the CITRIN (Comparison of InTRaosseous infusion systems in emergency medicINe)-Study
Source: PLoS One. 2015 Dec 2;10(12):e0143726. doi: 10.1371/journal.pone.0143726 (PMC4668027; doi:10.1371/journal.pone.0143726)
Supplement: S7 Table — (DOCX) [file pone.0143726.s007.docx]

**S7 Table: Cumulative volumes in ethanol-fixed body donors (not normalized).**

| **Number** | **EZ-IO Tibia** | | | | | |  | **EZ-IO Humerus** | | | | | |  | **FASTR** | | | | | |
| --- | --- | --- | --- | --- | --- | --- | --- | --- | --- | --- | --- | --- | --- | --- | --- | --- | --- | --- | --- | --- |
|  | **0 mmHg** | | | **150 mmHg** | | |  | **0 mmHg** | | | **150 mmHg** | | |  | **0 mmHg** | | | **150 mmHg** | | |
|  | **1 min** | **3 min** | **5 min** | **1 min** | **3 min** | **5 min** |  | **1 min** | **3 min** | **5 min** | **1 min** | **3 min** | **5 min** |  | **1 min** | **3 min** | **5 min** | **1 min** | **3 min** | **5 min** |
|  |  |  |  |  |  |  |  |  |  |  |  |  |  |  |  |  |  |  |  |  |
| 1 |  |  |  |  |  |  |  |  |  |  |  |  |  |  | 17.8 | 7.9 | 5.7 | 11.9 | 7.9 | 9.1 |
| 2 |  |  |  |  |  |  |  |  |  |  |  |  |  |  | 22.8 | 23.5 | 19.2 | 85.2 | 50.2 | 26.0 |
| 3 |  |  |  |  |  |  |  |  |  |  |  |  |  |  | 10.9 | 5.0 | 3.2 | 1.0 | 3.0 | 3.0 |
| 4 |  |  |  |  |  |  |  |  |  |  |  |  |  |  |  |  |  |  |  |  |
| 5 |  |  |  |  |  |  |  |  |  |  |  |  |  |  | 5.9 | 3.6 | 3.0 | 14.9 | 14.5 | 14.1 |
| 6 | 6.9 | 6.6 | 6.9 | 38.6 | 20.5 | 18.4 |  | 2.0 | 1.3 | 1.0 | 5.0 | 5.3 | 5.9 |  | 11.9 | 8.6 | 8.1 | 14.9 | 24.8 | 21.8 |
| 7 | 1.0 | 1.3 | 1.2 | 26.8 | 16.8 | 16.3 |  | 1.0 | 1.0 | 0.6 | 3.0 | 6.6 | 7.9 |  |  |  |  |  |  |  |
| 8 | 2.0 | 3.0 | 1.8 | 0.0 | 1.7 | 1.2 |  | 5.0 | 4.0 | 3.8 | 4.0 | 3.0 | 4.8 |  | 0.0 | 1.0 | 0.8 | 13.9 | 7.3 | 5.3 |
| 9 | 12.9 | 6.6 | 4.8 | 3.0 | 5.6 | 5.5 |  | 5.0 | 4.0 | 4.0 | 5.0 | 7.6 | 5.7 |  | 10.9 | 8.9 | 9.1 | 18.8 | 31.7 | 31.3 |
| 10 | 7.9 | 6.3 | 6.3 | 23.8 | 15.9 | 15.1 |  | 14.9 | 10.6 | 10.5 | 25.8 | 14.5 | 17.8 |  |  |  |  |  |  |  |
| 11 | 3.0 | 5.0 | 6.3 | 18.8 | 15.5 | 15.3 |  | 4.0 | 4.0 | 4.4 | 5.0 | 7.9 | 7.5 |  | 1.0 | 1.0 | 1.0 | 1.0 | 9.0 | 2.4 |
| 12 | 5.0 | 5.9 | 6.1 | 21.8 | 22.1 | 22.4 |  | 2.0 | 0.7 | 0.6 | 17.8 | 6.9 | 4.8 |  |  |  |  |  |  |  |
| 13 | 1.0 | 0.3 | 0.8 | 5.9 | 3.0 | 2.2 |  | 11.9 | 8.9 | 9.1 | 68.4 | 39.6 | 36.1 |  | 2.0 | 1.3 | 2.2 | 5.0 | 2.3 | 7.9 |
| 14 | 0.0 | 1.0 | 1.4 | 9.9 | 9.2 | 8.7 |  | 2.0 | 1.0 | 1.2 | 2.0 | 2.6 | 2.6 |  | 14.9 | 10.6 | 10.9 | 6.9 | 13.5 | 11.3 |
| 15 | 5.0 | 2.3 | 1.6 | 9.9 | 4.0 | 4.2 |  | 4.0 | 2.3 | 2.8 | 5.9 | 8.3 | 8.3 |  | 0.0 | 0.3 | 0.4 | 0.0 | 1.0 | 1.2 |
| 16 | 9.9 | 5.9 | 4.0 | 6.9 | 3.6 | 5.2 |  | 0.0 | 0.0 | 0.0 | 8.9 | 3.3 | 3.0 |  | 7.9 | 5.9 | 5.9 | 19.8 | 14.2 | 11.9 |
| 17 | 13.9 | 5.9 | 3.6 | 12.9 | 8.6 | 6.3 |  | 2.0 | 1.3 | 1.8 | 7.9 | 5.9 | 7.5 |  | 1.0 | 0.7 | 0.8 | 4.0 | 3.0 | 2.6 |
| 18 | 7.9 | 6.9 | 4.8 | 3.0 | 4.3 | 5.5 |  | 26.8 | 17.2 | 11.3 | 18.8 | 7.6 | 5.7 |  |  |  |  |  |  |  |
| 19 | 10.9 | 14.5 | 9.7 | 5.0 | 3.0 | 3.6 |  | 19.8 | 8.3 | 6.5 | 51.5 | 17.5 | 10.9 |  |  |  |  |  |  |  |
| 20 | 1.0 | 1.3 | 1.0 | 20.8 | 10.2 | 7.5 |  | 28.7 | 10.2 | 6.7 | 2.0 | 1.7 | 2.4 |  |  |  |  |  |  |  |
| 21 | 1.0 | 1.0 | 1.0 | 33.7 | 20.8 | 16.4 |  | 2.0 | 1.0 | 0.8 | 13.9 | 7.3 | 5.7 |  |  |  |  |  |  |  |
| 22 | 1.0 | 1.3 | 1.0 | 5.0 | 4.3 | 3.6 |  | 5.9 | 3.3 | 3.2 | 5.9 | 9.6 | 7.9 |  |  |  |  |  |  |  |
| 23 | 2.0 | 0.7 | 0.8 | 0.0 | 0.7 | 0.6 |  | 4.0 | 3.0 | 5.0 | 26.8 | 10.6 | 7.1 |  |  |  |  |  |  |  |
| 24 | 0.0 | 1.3 | 1.0 | 3.0 | 2.0 | 1.4 |  | 2.0 | 2.3 | 1.8 | 6.9 | 3.6 | 4.4 |  | 4.0 | 4.6 | 4.6 | 14.0 | 13.7 | 6.0 |
| 25 | 8.9 | 5.3 | 3.6 | 5.9 | 7.3 | 6.1 |  | 5.9 | 3.0 | 2.8 | 37.7 | 30.1 | 19.8 |  | 10.9 | 6.3 | 5.3 | 56.5 | 39.9 | 32.5 |
| 26 | 5.9 | 3.0 | 4.2 | 5.0 | 13.9 | 13.5 |  | 12.9 | 5.0 | 5.0 | 33.7 | 17.2 | 12.9 |  | 2.0 | 2.0 | 1.4 | 5.0 | 2.3 | 7.9 |
| 27 | 9.9 | 5.9 | 4.0 | 13.9 | 5.6 | 4.8 |  | 0.0 | 2.3 | 1.8 | 11.9 | 5.9 | 4.8 |  | 2.0 | 1.7 | 1.4 | 10.9 | 9.3 | 7.3 |
|  |  |  |  |  |  |  |  |  |  |  |  |  |  |  |  |  |  |  |  |  |
| **Mean value** | **5.3** | **4.2** | **3.4** | **12.4** | **9.0** | **8.4** |  | **7.3** | **4.3** | **3.8** | **16.7** | **10.1** | **8.8** |  | **7.4** | **5.5** | **4.9** | **16.7** | **14.6** | **11.9** |
| **Standard deviation** | **4.4** | **3.3** | **2.5** | **11.0** | **6.9** | **6.4** |  | **8.4** | **4.2** | **3.3** | **17.6** | **9.2** | **7.5** |  | **6.9** | **5.7** | **4.9** | **21.9** | **14.2** | **10.0** |
| ***p value inner group*** | ***0.001 - 0.013*** | | | | | |  | ***0.002 - 0.015*** | | | | | |  | ***0.02 - 0.054*** | | | | | |
| ***p value inter group*** | ***0.220 - 0.643*** | | | | | | | | | | | | | | | | | | | |
